# Determining correspondences between high-frequency MedDRA concepts and SNOMED: a case study

**DOI:** 10.1186/1472-6947-10-66

**Published:** 2010-10-28

**Authors:** Prakash M Nadkarni, Jonathan D Darer

**Affiliations:** 1 Geisinger Health Systems, Danville, PA, USA; 2 Center for Medical Informatics, Yale University School of Medicine, New Haven, CT, USA

## Abstract

**Background:**

The Systematic Nomenclature of Medicine Clinical Terms (SNOMED CT) is being advocated as the foundation for encoding clinical documentation. While the electronic medical record is likely to play a critical role in pharmacovigilance - the detection of adverse events due to medications - classification and reporting of Adverse Events is currently based on the Medical Dictionary of Regulatory Activities (MedDRA). Complete and high-quality MedDRA-to-SNOMED CT mappings can therefore facilitate pharmacovigilance.

The existing mappings, as determined through the Unified Medical Language System (UMLS), are partial, and record only one-to-one correspondences even though SNOMED CT can be used compositionally. Efforts to map previously unmapped MedDRA concepts would be most productive if focused on concepts that occur frequently in actual adverse event data.

We aimed to identify aspects of MedDRA that complicate mapping to SNOMED CT, determine pattern in unmapped high-frequency MedDRA concepts, and to identify types of integration errors in the mapping of MedDRA to UMLS.

**Methods:**

Using one years' data from the US Federal Drug Administrations Adverse Event Reporting System, we identified MedDRA preferred terms that collectively accounted for 95% of both Adverse Events and Therapeutic Indications records. After eliminating those already mapping to SNOMED CT, we attempted to map the remaining 645 Adverse-Event and 141 Therapeutic-Indications preferred terms with software assistance.

**Results:**

All but 46 Adverse-Event and 7 Therapeutic-Indications preferred terms could be composed using SNOMED CT concepts: none of these required more than 3 SNOMED CT concepts to compose. We describe the common composition patterns in the paper. About 30% of both Adverse-Event and Therapeutic-Indications Preferred Terms corresponded to single SNOMED CT concepts: the correspondence was detectable by human inspection but had been missed during the integration process, which had created duplicated concepts in UMLS.

**Conclusions:**

Identification of composite mapping patterns, and the types of errors that occur in the MedDRA content within UMLS, can focus larger-scale efforts on improving the quality of such mappings, which may assist in the creation of an adverse-events ontology.

## Background

The Systematic Nomenclature of Medicine Clinical Terms (SNOMED CT) [1], originally developed by the College of American Pathologists, and now managed by the International Health Terminology Standards Development Organization (IHTSDO) has been repeatedly demonstrated to be the most comprehensive single-source controlled vocabulary with respect to clinical content coverage[2-7]. Consequently, there are efforts at several levels toward making SNOMED CT the basis for encoding of clinical documentation.

Adverse events following therapeutic interventions, e.g., pharmaceutical preparations and medical devices, are an important and often preventable, cause of morbidity and mortality in patients. While some Adverse Events are discovered through preclinical testing and clinical trials prior to drug or device approval, the vast majority are discovered during post-marketing safety surveillance simply because of the much larger number of patients who are exposed to the agent over longer periods of time. With the push towards wider deployment of electronic medical records (EMRs), the EMR is likely to become an increasingly important source of Adverse Event discovery. Several efforts, notably by Carol Friedman's group at Columbia University, have been directed at using natural-language processing (NLP) techniques to determine the feasibility of detecting Adverse Events in clinical text [8,9]. If such efforts prove successful, it may become possible to enable automated or semi-automated Adverse Event detection.

The US Federal Drug Administration's Adverse Events Reporting System (AERS)[10] supports post-marketing surveillance. Reporting to the AERS is voluntary in the US for primary health care providers and others, such as patients, patient relatives, and lawyers. If, however, such individuals have reported a problem to the agent's manufacturer, the latter is legally required to send the report to the FDA. Within the US and internationally, the controlled vocabulary that forms the basis of adverse event reporting, both for post-marketing surveillance as well as for drug agency-regulated clinical trials, is the Medical Dictionary for Regulatory Activities (MedDRA) [11], developed by the International Conference on Harmonization (ICH). MedDRA includes concepts not only for Adverse Events but also for the therapeutic indications for which the medication/device was employed. The "Adverse Events" also include errors in prescribing, dispensing and formulation of therapeutic agents, interactions of drugs with other drugs or food, as well as errors that were intercepted before they reached the patient. Both SNOMED CT and MedDRA are components of the Unified Medical Language System (UMLS) [12], a compendium ("meta-thesaurus") of a large number of biomedical vocabularies that is distributed by the National Library of Medicine (NLM).

If SNOMED CT becomes the basis for encoding clinical documentation, NLP software that assists the encoding of narrative text into SNOMED CT concepts may eventually be bundled ubiquitously with EMR software. Much NLP software, e.g., GATE [13] and MedLEE [14] is implemented using a pipeline of individual modules, each of which is specialized for a particular sub-task, e.g., section and sentence segmentation, part-of-speech tagging, and named-entity recognition. Rather than creating special-purpose software to generate MedDRA concepts directly from raw clinical text, vendors may find it far simpler computationally to add a MedDRA-generation module at the end of the existing pipeline, to map SNOMED CT concepts into MedDRA equivalents. Such a module will need to utilize a cross-mapping between the SNOMED CT and MedDRA vocabularies. It is therefore essential that these mappings be both comprehensive and of high quality.

In this paper, we describe the issues that were encountered in an exercise aimed at creating a map between MedDRA and SNOMED CT, focusing on high-frequency MedDRA terms for adverse reactions and therapeutic indications in one year's worth of AERS data, which was recorded between July 1, 2008 and April 30, 2009. The motivation for this work is that, as illustrated subsequently, the existing mappings between MedDRA and SNOMED CT are limited and that there are a variety of integration errors in the mapping of MedDRA to UMLS, which is the starting point for our efforts.

Our research objectives are stated below:

• To identify aspects of MedDRA that complicate the process of mapping MedDRA concepts to SNOMED CT.

• Through the attempted mapping of high-frequency unmapped MedDRA concepts to SNOMED CT:

○ To categorize patterns that allow composition of MedDRA concepts using SNOMED CT concepts;

○ To identify possible lacunae in SNOMED CT coverage of the adverse event domain;

○ To identify errors where a one-to-one mapping of an unmapped MedDRA concept to a SNOMED CT concept becomes apparent on human inspection, but has not been created in the UMLS.

• To identify patterns in the integration errors in the mapping of MedDRA to UMLS.

### An Overview of Terminological Issues related to MedDRA

Any attempt to cross-map between SNOMED CT and MedDRA must commence with an understanding of the strengths and limitations of each vocabulary. SNOMED CT is designed along sound ontological principles. Due to vigorous curation efforts, its content continues to improve with each release. However, the vast size of SNOMED CT - currently around 300 K active concepts - limits the possibility of using all of its content as an "interface terminology" [15], i.e., one intended to be employed directly by end-users. The SNOMED CT documentation recommends the creation of subsets to serve special purposes such as coding in clinical sub-domains.

For the purpose of Adverse Event capture, MedDRA has the relative advantage of brevity (65 K terms overall) and it is conceivable that the equivalent SNOMED CT concepts would form an "Adverse Event subset". However, MedDRA's design limitations, described in [16-18], which are a consequence of its emphasis on classification, complicate the mapping process and mitigate its usefulness with respect to eventual query and analysis of AERS data at various levels of granularity[19,20]. To be fair, MedDRA's creation antedates the current emphasis on integrating ontological principles into controlled vocabulary design, as in the case of SNOMED CT.

• Sophisticated controlled vocabulary designs such as implemented in SNOMED CT and UMLS model collections of concepts as graphs with an approximately hierarchical structure. There can be as many levels of hierarchy as are considered appropriate: the number of levels tends to be more for biomedical sub-domains that have been studied for longer periods of time.

By contrast, MedDRA content is artificially constrained to a five-level hierarchy: System Organ Classes, High Level Group Terms, High-level Terms, Preferred Terms and Lower Level Terms. The FDA AERS records indications and adverse reactions at the Preferred Term level; there are about 18 K Preferred Terms in MedDRA currently.

• Individual concepts in a vocabulary should be allowed to descend from more than one parent if necessary: for example, tuberculosis of the spine is both a kind of tuberculosis and a disorder of the spinal column. In MedDRA, this flexibility is artificially limited: Preferred Terms may descend from more than one Higher-level Term, but a given Higher-level Term or Higher-level Group Term may descend from only one SOC. This leads to difficulties in formulating queries.

• In MedDRA, there is no semantic consistency in the relationship between a Lower-level term and a Preferred Term. Some Lower-level terms are lexical variants or synonyms of Preferred Terms - e.g., "cataract, lenticular" is an Lower-level term for the Preferred Term "cataract" - but others are more specific concepts that should really be coding concepts in their own right - e.g, "diphtheritic myocarditis" is an Lower-level term related to the Preferred Term "diphtheria". Determining the precise semantics of the relationship requires human interpretation. In many cases (such as the cardiac arrhythmias), the Lower-level terms for a given Preferred Term, if looked up in SNOMED CT, may be discovered to have hierarchical relationships among themselves, but these cannot be modeled in MedDRA because a concept "lower" than an Lower-level term is not permitted. The FDA AERS public data provides Adverse Event details at the Preferred Term level only; finer-level detail that may be clinically relevant, in the case of more specific concepts, is not accessible.

• MedDRA falls short with respect to several of Cimino's well-known controlled-vocabulary desiderata[21] in addition to the problems of hierarchy discussed above:.

○ MedDRA is a *non-compositional *vocabulary: that is, it is not possible to combine concepts to form new concepts using specified operators. All concepts used in MedDRA are pre-coordinated, i.e., formed by synthesizing a phrase that combines individual concepts, where the semantics of the concept must be determined by human inspection of the terms associated with the concept. A large number of such pre-coordinated concepts combine a laboratory test, e.g., "bone alkaline phosphatase", with a "qualifier" attribute that describes the result of the test qualitatively: e.g., normal, abnormal, increased, decreased, positive negative.

○ MedDRA uses *semantically ambiguous *qualifying phrases such as "other/not otherwise specified" and "not elsewhere classified" in many terms. Such concepts are hard to interpret because they are valid only within a single vocabulary, and even here, are based on a criterion of exclusion that is not time-invariant: more categories may subsequently be added, so that the meaning of the concept drifts.

• Certain pre-coordinated concepts in MedDRA (albeit concepts that do not appear to be used in actual AERS data) are not clinically meaningful;. An example category is "(substance) low", where the substance may be lead, cadmium, beryllium, cyanide and several other toxic substances, whose normal level in any tissue should be zero: the concept of a "low" level is invalid. (There is a separate concept "lead decreased" in MedDRA: this concept, which refers implicitly to the presence of a previous measurement, can be meaningful when used to describe the effect of chelation therapy in lead poisoning.)

### Existing Mappings between MedDRA and SNOMED CT

The content of the UMLS's MRCONSO table, which contains mappings of concepts in individual source vocabularies to concepts in the UMLS, can serve as the starting point for mapping vocabularies to each other. The overlap between all of the MedDRA concepts in UMLS and SNOMED CT concepts is around 40%: however, 58% of preferred terms, the most useful part of MedDRA, map to SNOMED CT, as reported by Bodenreider [22].

Our inspection of the MedDRA content that was mapped to UMLS, however, revealed numerous problems that we describe in the Discussion section. In addition, mapping between concepts in a pre-coordinated vocabulary like MedDRA and a vocabulary that allows composition (SNOMED CT) is not guaranteed to be one-to-one. For example, most laboratory-related MedDRA concepts that conflate the test measurement with the qualitative result do not occur in SNOMED CT, but need to be composed by combining the concept of the measurement with a SNOMED CT "qualifier" concept like "increased" or "elevated". We later consider, in the Discussion, some of the analytical issues that this approach raises.

## Methods

The 2009AA release of the UMLS and Jan'09 release of SNOMED CT, obtained from the UMLS Knowledge Server, were used for this work.

### Source of Adverse-Event Frequency Data

Version 11.1 of MedDRA contains 18,209 Preferred Terms. In order to focus our mapping efforts, we assumed that every Preferred Term would be unlikely to occur in actual FDA AERS data, and that a relatively modest proportion of Preferred Terms would account for the majority of actual AERS records. Data for the study was therefore obtained from the FDA AERS download site [23] in order to identify the Preferred Terms that occurred with high frequency. We decided to select those Preferred Terms that collectively accounted for more than 95% of the AERS therapeutic indications and adverse event records: the 95% threshold has been used in other initiatives, such as the NLM's SNOMED CT CORE Subset [24].

### Data Set Characteristics

The characteristics of the data set are given in Table 1 below.

**Table 1:** Characteristics of the FDA AERS Data Set used to identify high-frequency concepts

	Therapeutic Indications Data	Adverse Events Data
A. Number of records in data set (each record contains 1 Preferred Term)	850 K	1.19 M

B. Unique Preferred Terms in data set	5,540	10,304

C. Number of unique Preferred Terms already mapped to SNOMED CT in UMLS	3864 (69.7%)	6,409 (62.2%)

D. Number of unique Preferred Terms accounting for >95% of records ("high-frequency" Preferred Terms)	834 (15.1%), accounting for 60+ records each	2,871 (27.9%), accounting for 32+ records each

E. Number of high frequency Preferred Terms already mapped to SNOMED CT in UMLS	693 (83.1%)	2,226 (77.5%)

F. Number needing manual mapping (D-E)	141	645

### Mapping of Unmapped Preferred Terms

The first author developed a SNOMED CT browser/concept-searcher using Microsoft SQL Server 2008 to host the Jan '09 SNOMED CT content, with a stored-procedure library written using Visual Basic .NET 2008, and a browsing front-end created using Microsoft Access 2003. The SNOMED CT content was augmented with single-word synonym content from the Unified Medical Language System (i.e., all terms in UMLS for a given SNOMED concept where one of the terms comprises a single lexical token). This software will be made available freely on request to the first author; however, its use is not critical to this work, and alternative concept-searching software, such as the popular CLUE browser http://www.clininfo.co.uk, could have been used.

The user specifies search terms either by pasting the MedDRA term into a text box, or entering keywords manually. (The software can also run in batch mode: this mode was used to fetch matching candidates into a table for subsequent manual inspection and selection.)

Concepts matching one or more words in the search phrase are returned using the well-known "Term Frequency * Inverse Document Frequency" (TF*IDF)[25] approach for relevance-ranking of search results. The concept-searching process eliminates stop-words from a search phrase (very common words in a language that have minimal search value) using the PubMed stop-word set[26]. The remaining words in the phrase are lemmatized [27], i.e., conversion to root forms (lemmas) by eliminating variations in tense and person using the program *morph*, part of the well-known Wordnet thesaurus/software [28]. Lemmatization can sometimes yield more than one lemma from a given word e.g., the lemma of "leaves" can be "leaf" or "leave", depending on whether "leaves" is a plural noun or a verb. Lemmatization results in query expansion because the lemmatized words are searched against a lemmatized word index. Further query expansion is performed electronically using a manually created list of Greco-Latin/common-word and other equivalences (e.g., hepatic/liver, gastric/stomach, neoplasm/tumor).

The IDF*TF approach is most appropriate for circumstances where a single concept in SNOMED CT is likely to contain all or most of the words in the MedDRA term. However, a preliminary search of unmapped MedDRA terms had revealed a large number of terms where post-coordination would be necessary, e.g., when a lab test is combined with a single-word qualifier. Here, using the IDF*TF approach to retrieving every SNOMED CT concept that contains at least one word in the search term would return a prohibitive number of concepts. Therefore, the software additionally tries to simultaneously locate SNOMED CT concepts with synonymous terms corresponding to single words in the search phrase. This is useful for terms such as "CYTOMEGALOVIRUS CHORIORETINITIS" and "HAEMATOCRIT ABNORMAL".

## Results

The results of the mapping process are shown in Table 2 below.

The details of the mappings are available in the online data supplement (Additional File 1) that accompanies this paper. We summarize the highlights of the mapping below: when we use a concept ID of the form Cnnnnn, we refer to the UMLS CUI.

**Table 2:** Results of the mapping process

	Indications Preferred Terms	Adverse Event Preferred Terms
*Total number of Preferred Terms requiring manual mapping (Table 1, row F)*	*141*	*645*

Preferred Terms not in SNOMED CT and not composable from existing SNOMED CT concepts	7	46

Preferred Terms mapping to a single SNOMED CT concept (missed synonyms)	45	195

Preferred Terms composable from two SNOMED CT concepts	87	373

Preferred Terms composable from three SNOMED CT concepts	2	31

### Compositional Patterns

As expected, many MedDRA Preferred Terms had to be composed using more than one SNOMED CT concept: other than the "missing concepts" below, none required more than three SNOMED CT concepts to compose. The following types of composite forms were encountered:

<observation of laboratory test>

<qualitative result>;

<organism> < "infection"/"bacteremia"/"sepsis">;

<malignancy> < "stage"/"recurrent"/"metastatic">;

<pharmacological-action> < "therapy"/"supportive care">;

<disease> "prophylaxis";

<body part> "pain"/"inflammation"/"injury";

<organ system> "toxicity".

(Strings are denoted in quotes, while unquoted phrases refer to a concept category. Slashes refer to alternatives within a pattern, while carets are used to denote a group within a pattern.)

### Concepts Missing in SNOMED CT

Some concepts were missing in SNOMED CT. Among Adverse Events, errors in prescription or administration of a drug are under-represented in SNOMED CT, and concepts such as therapy cessation, device leaks/breakage, off-label use, product contamination, tampering, counterfeiting and quality issues may be expected to be missing. Omissions of clinical coverage include multiple drug resistance (to antibiotics), propofol infusion syndrome, compulsive shopping behavior, and intrauterine device migration, paternal drugs affecting fetus. Among Indications, concepts not expected to be in SNOMED CT are "Drug use for unknown indications" (the highest-frequency indication); "evidence-based treatment" and "unevaluable event". Clinical coverage omissions are breakthrough pain (seen in patients receiving narcotics for terminal illness) and "bone marrow conditioning regimen". Some of the indications terms are also Adverse-Event Terms (paternal drugs affecting fetus, off-label use).

The common concept of post-procedural complications is not represented in SNOMED CT and needs to be composed. A family of MedDRA Preferred Terms that required three SNOMED CT concepts to map was the qualitative results of therapeutic drug monitoring. These had to be expressed using SNOMED CT concept 365750008 ("Finding of therapeutic drug level"), the drug family (e.g., anticonvulsant), and the qualitative result.

### Unmapped MedDRA concepts matching single SNOMED CT concepts

What was surprising was that about 30% of the "unmapped" Preferred Terms for both indications and Adverse Events were found to map to single SNOMED CT concepts, indicating insufficient checking of these Preferred Terms during the MedDRA-UMLS curation process prior to characterizing them to distinct concepts in UMLS. This missed synonymy results in redundancy in UMLS content. Many of these were synonyms (e.g., dysphemia C1096340 = stuttering C0038506); word variants (feeling guilty C0877289 = feeling guilt (finding) C0018379); abbreviations (hip dysplasia C1328407 = congenital acetabular dysplasia (disorder) C0431952 - hip dysplasia, by definition, is never "acquired").

The problem of missed synonymy in the UMLS was identified by Hole and Srinvasan in a 2000 paper [29] and is admittedly a difficult one to solve. As we emphasize shortly, the concept redundancy problem is much more prominent for MedDRA's lower-level terms, many of which are synonyms for existing concepts rather than concepts in their own right.

## Discussion

### Issues with the MedDRA content in UMLS

The process of integrating a source vocabulary into UMLS involves identifying the source-vocabulary concepts that correspond to existing UMLS concepts. Concepts where such correspondences are not found are designated as new concepts that will be assigned UMLS Concept IDs. However, the NLM performs limited curation, relying partly on automated methods, and to some extent on the diligence and knowledge of source-vocabulary curators of the individual source vocabularies for this process. In any case, errors in the original MedDRA content can propagate to UMLS. Several types of errors can be detected in the source-vocabulary-to-UMLS mapping process, but their detection typically requires content expertise and manual inspection; some of these, indicated with a double asterisk, are due to undetected problems in the original MedDRA content.

• Missed Synonymy: Many concepts in MedDRA become new, duplicated concepts in UMLS: We have provided some examples earlier. Additional causes of missed synonymy include British vs. American spellings: For example, both "Aluminium" (IUPAC/British) and "Aluminum" (US) occur in phrases involving the metal. For example, we have the MedDRA derived "Blood aluminium (& level (& serum))" C1578975" and SNOMED CT derived "Serum aluminum level" C1318288.

• Improper Designation of Synonymy: Many pairs of distinct concepts are treated incorrectly as though they were synonyms.

○ ** In the case of qualitative laboratory results, MedDRA does not appear to distinguish "increased" from "high", or "decreased" from "low": it treats these pairs (loosely) as interchangeable synonyms. Thus, there are the concepts "Digoxin level high" C0581164, and "Digoxin level decreased" C0920128), but there is no concept "Digoxin level increased". Strictly speaking, "increased" and "decreased" imply comparison to a previous value, which has relevance to the therapeutic drug monitoring that is employed for Digoxin, while "high" and "low" refer to values above or below normal/therapeutic ranges. SNOMED CT differentiates between "high" (synonym "elevated") and "increased" by placing them in different sub-hierarchies.

○ ** MedDRA tends to use "carcinoma" as a synonym for malignancy/cancer, whereas the term strictly refers to a malignancy of ectodermal or endodermal origin, as opposed to a "sarcoma" that derives from mesoderm. Thus, MedDRA has the concept of "gastrointestinal carcinoma", but does not record the (admittedly rarer) concept of gastrointestinal sarcomas, or the general concept "gastrointestinal cancer" as a distinct concept.

• ** Certain medical phrases that commonly occur in concepts are used loosely, so that they become new concepts in UMLS that, on closer inspection by someone with a background in laboratory medicine, would turn out to be invalid. For example, MedDRA's use of "Blood" encompasses serum, plasma and blood as the sample source, while SNOMED CT's use is precise. So the MedDRA-derived "blood prolactin" C0853129 does not exist as a SNOMED CT concept (whole blood is not used for prolactin assays, so that the concept of a "blood prolactin assay" is invalid), though "plasma prolactin" C0857712 does.

• ** The unclear relationship between MedDRA preferred terms and lower-level terms results in spurious concepts in UMLS and inaccurate relationships. For example, In the UMLS-MedDRA content, "antimony normal" C0861045 is treated as a distinct concept from "blood antimony normal" C0855863. Interpreted in isolation in UMLS, the former concept is inherently ambiguous (it does not specify the tissue that was sampled). Inspection of the details of the two concepts, however, reveals that in MedDRA, "antimony normal" is a lower-level term corresponding to the preferred term "blood antimony normal". This should indicate that it is a synonym: it cannot be a more *specific *type of antimony measurement, since "any" tissue is more *general *than "blood". Note that, this concept is also meaningless clinically: organic antimonials are used only in the treatment of schistosomiasis and leishmaniasis, and the "normal" level is zero.

• Sometimes, MedDRA concepts are matched to the wrong UMLS concept. An example is "Chemistry", which is matched to C0007996, which refers to the science (like physics and biology). The term "chemistry", however, is known to be a homonym (a word with multiple, separate meanings) in English, and an inspection of the MedDRA-derived relationships of this concept in UMLS reveals that it is an Lower-level term corresponding to the Preferred Term "Laboratory Procedure", indicating that it is really a synonym (a non-preferred, abbreviated form) for "Serum Chemistry Test". Many scientific phrases are semantically ambiguous in isolation, and the accepted way to address these is by the use of descriptive concept definitions (a mammoth curatorial task where all existing terminologies fall short) and/or the labeling of certain terms as ambiguous (a process followed by SNOMED CT and UMLS). MedDRA, however, lacks concept definitions entirely, and there is no recognition or labeling of term ambiguity within its content: the meaning of a term must often be inferred from its position in a hierarchy. Consequently, errors of this kind are less likely to be detected.

As a consequence of the above issues, the existing mapping in UMLS between SNOMED CT and MedDRA is problematic and needs to be carefully checked. In particular, the excessive incorporation of synonymous MedDRA Lower-level terms as new concepts in UMLS vitiates UMLS's intended role as a "Rosetta stone".

### Dealing with MedDRA Concepts based on Laboratory Parameters

As discussed previously, to map a large number of MedDRA concepts based on laboratory parameters to SNOMED CT equivalents, one needs to combine the concept of the laboratory test measurement with a "qualifier" concept describing the qualitative result of the result, e.g., normal, high or low. This approach may satisfy the objective of mapping, but it does not address the problem of analyzability of the resulting SNOMED CT-encoded data.

• In the EMR, laboratory data is typically recorded in structured form rather than reproduced in the unstructured clinical narrative. The EMR's laboratory-data subsystem has standard methods of determining whether a given result is in the normal range, based on reference/laboratory standards and the patient's age and sex: the clinician can additionally bring information on other physiological states such as pregnancy to bear on the result. The task of generating appropriate MedDRA codes from structured laboratory data is algorithmically far simpler than trying to do the same with narrative text: such text may contain only the numerical values of the lab parameters, whose names are often reported in abbreviated form without mention of reference range or even units.

• Laboratory measurements are currently not modeled in detail in SNOMED CT: they are mostly "primitive" concepts rather than fully defined. This makes such mappings less useful than mappings to, say, clinical diagnoses.

• It is well known that "Qualifier" concepts, such as used to describe qualitative results, are currently one of the least developed aspects of SNOMED CT: Rector and Brandt [30] show that for many circumstances where they are used, more formal approaches would be preferable. They do not allow even limited electronic reasoning of the kind that is useful for Adverse Events - for example, that for certain tests, "abnormal" and "high" are synonymous, while for others (such as serum electrolytes and hormone levels), both "high" and "low" are abnormal results. This is partly because of the lack of any detailed computable semantics associated with individual qualifiers, which makes it easy to misuse "elevated" when "high" is intended, for example.

The use of ordinal domains [31] -sets of permissible values for a laboratory-result concept that can describe a result qualitatively, based on a quantitative definition of normal - allows straightforward electronic reasoning. Such reasoning is implemented routinely by laboratory reporting systems that place an "H(igh)" or "L(ow)" against a numeric value, and would be extremely difficult if a purely qualifier-based approach to reasoning were adopted.

The lack of ordinal-value support is a common problem in ontologies that is not limited to SNOMED CT. There is no intrinsic reason, however, why such support - a standard aspect of database/analytics knowledge representation for over three decades - cannot be systematically integrated with ontologies in knowledge domains where such support is called for. The latest incarnation of the Web Ontology Language, OWL 2, seems to be moving in this direction, with improved support for mathematical operations as well as more expressive constraints[32]. The SNOMED CT concept model for observables is under active revision, but its current draft version (0.03) does not provide support for ordinal-value representation.

### Toward a Comprehensive Ontology of Adverse Events

In this section, we discuss the implications of the work described.

Even if NLP techniques are able to match concepts in clinical text to Adverse Events with high accuracy, they do not address the problem of eventually aggregating the results in meaningful ways for analysis. In clinical narrative, certain concepts may be specified explicitly by the caregiver, while at other times they may be unstated, and their presence must be inferred from their known relationship to other concepts that occur in the narrative.

For example, in the paper of Wang et al (the Columbia group) [8], phrases encountered in various patients undergoing treatment with bupropion (used as an anti-depressant as well as for smoking cessation) included "extrapyramidal sign", "stiffness", and "motor retardation". The authors do not recognize that the latter two symptoms are part of the extrapyramidal syndrome when occurring together (though each in isolation can have other causes), and an analysis that treated these terms as isolated entities would under-count the extrapyramidal findings. (MedDRA contains information about sets of individual findings that, when co-occurring, suggest specific syndromes. This information, the Standardized MedDRA Queries (SMQs), was created to facilitate data mining, and there is already an SMQ for Extrapyramidal Syndrome. The authors did not use SMQ data, which is available in the UMLS Rich-Release format, to attempt to improve the accuracy of their results.)

The availability of high-quality adverse events information content, which reliably records relationships between Adverse-Event concepts more comprehensively than MedDRA does currently, can improve the quality and productivity of Adverse-Event data analysis and data-mining, by minimizing the effort involved in ad-hoc creation of aggregate groupings that need to be replicated by every research group working with such data. The availability of Gene Ontology [33] has had such benefits in the gene-expression microarray field, by facilitating the summarization of signals from thousands of genes into fewer, more readily interpretable categories using gene-family and pathway labels.

An Adverse-Events ontology does not currently exist. The work reported in [34] has, however, explored the manual construction of such an ontology for the limited domain of hepatitis. MedDRA's design is not sufficiently robust to serve as the foundations of such an ontology, though its Preferred Terms may serve as the nodes of interest, and the SMQ content is also essential. The subset of SNOMED CT that maps to MedDRA (along with intermediate SNOMED CT concepts in the network that are discovered in the cross-mapping process) is more suitable for the ontology scaffolding. The work described in [35,36] has explored and confirmed this possibility for limited sub-domains such as "hemorrhage": this work has identified the need for new inter-concept relationship types (attributes) that are specific to the adverse-event domain. The work of Bodenreider [8] has also explored such issues, employing cross-mapping from MedDRA to SNOMED by automated approaches to actually quantify the intermediate-level SNOMED CT concepts.

The major use-case for a standardized, validated and freely available adverse-event ontology is to serve as the basis for eventually encoding existing knowledge about drug or device-related adverse events. Currently, most commercially distributed drug databases record adverse effects as narrative text reproduced from the FDA package insert: where information is encoded, International Classification of Diseases (ICD) diagnosis codes are used, so that the numerous (non-billable) subjective or objective findings that are not full-fledged diagnoses are not represented. The variability of narrative text makes it difficult to answer the question: given the presence of a particular clinical finding, which of the medications that the patient is taking could be responsible, and what is the likelihood (expressed on an ordinal scale) of individual medications contributing? It is clear that many of the issues involved in knowledge representation (including representation of SMQs) go beyond the strictly "ontological" - indeed, existing ontology-modeling tools and paradigms may prove a poor match for several aspects of the modeling of the adverse-event component alone. The effort required will be vast, requiring the resources of national/international organizations, but we hope that the initial exploration described here will provide signposts to potential minefields.

### Limitations of the present work

The limitations of this work include the following:

• The mappings that we have performed may contain errors, or be disputed by others. This paper is concerned primarily with the discovery of composition patterns as well as the MedDRA quality issues that were discovered during the attempted mapping. We have, however, provided a downloadable online appendix containing the mappings (as an Excel spreadsheet) as a companion to this paper.

• The numerous existing mappings of MedDRA to SNOMED CT in UMLS were not checked exhaustively: it is likely that there may be errors in addition to the ones that were caught in our exploration.

• Mappings between any two vocabularies with different underlying designs and incomplete overlap are necessarily directional. Our work only considers mapping from the non-compositional, smaller, MedDRA to the compositional, larger SNOMED CT. The reverse scenario - mapping SNOMED CT-encoded content from the clinical narrative to MedDRA concepts - has not been addressed. This can be challenging in cases where SNOMED CT concepts are recognized in clinical text by automated methods: such methods by their nature tend to discover simple, "primitive" concepts rather than highly composite ones (especially those that involve negation). Also, certain MedDRA concepts such as "wrong technique in drug usage process" must be inferred from the narrative, because they are rarely if ever explicitly stated as such.

## Conclusions

The design of MedDRA, in particular the failure to distinguish between lower-level terms that are synonyms of preferred terms as opposed to those that are distinct but finer-grained concepts, poses problems for the MedDRA content in UMLS. In addition, the existing integration approach has resulted in a significant proportion of duplicate concepts being added to UMLS. This content needs a detailed audit, because most researchers use UMLS as the source of MedDRA content rather than paying a significant subscription fee to the MedDRA Maintenance and Support Organization.

One challenge in other groups' building on the work described here is the currently very limited deployment of SNOMED CT in EMRs: a combination of factors such as licensing issues, vocabulary size, vendor unfamiliarity with ontological principles, and vendor inertia are responsible: in the USA, the last can be partly attributed to the fact that, while abstraction of clinical text for ICD-9 diagnoses is mandated for billing and reimbursement purposes, it is harder to justify the software development to assist SNOMED CT-encoding of clinical encounters (which is also much more extensive than diagnoses or procedure encoding) on a purely economic basis. Also, while SNOMED CT has the advantage of allowing post-coordination, error-free post-coordination either by local curators or clinicians requires intuitive and responsive software that is driven by a machine-readable concept model (an area of active development by the IHTSDO). We are optimistic, however, that the widespread adoption of SNOMED CT across EMRs will happen within a reasonable timeline. Once this happens, application of the SNOMED CT infrastructure to areas such as adverse-event capture will become a reality.

## Competing interests

None: the software whose use is described in this paper will be made freely available on request.

## Authors' contributions

PMN designed the software. Both PMN and JDD performed the mappings. JDD served as the authority on their accuracy and corrected them if necessary. All authors read and approved the final paper.

## Availability of Software

The software used by the author to perform the mapping will be made freely available on request.

## Pre-publication history

The pre-publication history for this paper can be accessed here:

http://www.biomedcentral.com/1472-6947/10/66/prepub

## Supplementary Material

Additional file 1 **Mappings of SNOMED Terms to MedDRA equivalents**. There are three worksheets in this workbook. The READ ME worksheet documents the contents of the other two worksheets. The sheet MedDRA_AEs records mapping of adverse event preferred terms to SNOMED concepts. The sheet MedDRA_indications records mappings of therapeutic indications to SNOMED concepts. Click here for file
